# The Physician as a Sanitarian*Address to the graduating class, Texas Medical College, Galveston, May 12, 1900, by Prof. W. S. Carter, M. D., of the Faculty.

**Published:** 1900-07

**Authors:** W. S. Carter


### TEXAS MEDICAL JOURNAL.

AUSTIN, TEXAS.

ESTABLISHED JULY, 1885.

PUBLISHED MONTHLY.—SUBSCRIPTION $1.00 A YEAR.

VOL. XVI. AUSTIN, JULY, 1900. No. 1.

### Original Contributions.

For the Texas Medical Journal.

### The Physician as a Sanitarian.*

*Mr. President, Members of the Board of Regents and of the Faculty, Members of the Graduating Class, Ladies and Gentlemen:*

The never-ending cycle of years has once more turned round, and it falls my turn to address you. On behalf of the faculty, I congratulate you on the splendid effort you have made in preparation and welcome you into the profession in which you have chosen to perform your life-work. This is an event that comes but once in a life-time, and is always attended with the sorrow of departure and the joy of expectation.

How fitting that this occasion should be called commencement—the commencement of your professional careers. What you have accomplished in the few years you have been with us has been but preparation to fit you for that work. Do not delude yourselves with the idea that the period of study is past. No error could be more fatal, especially to the physician. The mere attainment of the degree does not mean that your intellectual labors cease. If you aspire to success in your profession you will continue your studies with new effort, especially in the early years of your practical life.

It is, indeed, a unique opportunity to speak just at this time, as we are about to enter upon a new century. Such a time naturally suggests a retrospect—some review of the achievements and progress of the dying century. We are also entering upon a new era in med-

*Address to the graduating class, Texas Medical College, Galveston, May 12, 1900, by Prof. W. S. Carter, M. D., of the Faculty.

icine, and the line which marks it from the past is sharper than the changing of the centuries. It is a rare privilege to address the first graduates who have pursued the four years' course in medicine, not only in this State and university, but in this great southland. It is all the more to your credit that you should have elected such a course at a period when it was possible to get a degree elsewhere in less time, and in a State which, although it is most liberal in providing free professional education, is also most negligent in failing to protect the lives of its citizens against all sorts of quacks. Not only are men who are totally unfit permitted to practice medicine, but this State also gives a charter to a bogus institution for granting diplomas to such men. Although you are small in numbers you represent a very great gain in power.

Medical teaching has undergone radical changes in the last few decades of the closing century. In 1853, that great leader in medicine in this country, Dr. George B. Wood, with his characteristic eloquence, called attention to the urgent need of graded courses and of more clinical instruction, with less didactic teaching in the medical schools of the United States. It took twenty years for these suggestions to be put into operation. From that time rapid changes have been made in medical teaching in this country. In the last two decades, however, greater progress has been made in medicine than in the first eight decades of the century. With the advent of bacteriology more exact methods of investigation of disease have come into use in all the medical sciences, and the way of teaching all subjects has rapidly changed from the theoretical to the practical. Before this period but two years were required to prepare men for the practice of medicine by all the colleges of this country, impossible as that may seem at the present time. Until very recently but three years were required, but now four years are demanded by all the leading schools of the country. This great change in the preparation of men for the practice of medicine indicates that the training must be much more comprehensive and that new demands are made upon them; that more is expected of them and they are better prepared than in former times.

It may not be out of place at the present time, when we are hearing so much about national expansion, to speak of one of the possibilities presented in this expansion of medicine, and I have selected for my theme "The Physician as a Sanitarian."

It has been stated that although surgery has advanced greatly as a result of the knowledge gained from bacteriology, medicine has profited comparatively little from it. While in the cure of disease our earlier expectations may not as yet have been fully realized (with the exception of diphtheria), yet along the line of prevention possibilities are presented which are fully as great as in surgery. Preventive medicine probably offers a more brilliant future than any branch of science.

In former times four-fifths of all deaths were due to preventable diseases. At that time, however, the causes and methods of prevention were unknown. Conditions are different now. Bacteriology has given us accurate knowledge of the cause, the way of spreading and the certain means of preventing most of the contagious diseases. Even now, when the mortality has been reduced to about one-fourth of what it formerly was, still one-third of all deaths are due to preventable diseases.

Public medicine or sanitary science has for its object the prevention of diseases. The condition of this country in general sanitation is about a quarter of a century behind that of England. The urgent need of public medicine becomes more and more apparent every year, but is only beginning to be recognized. When this great country was more sparsely settled there was not the same necessity for it as at present; but with the country becoming more thickly populated, the cities growing rapidly and becoming crowded, more attention must be paid to public sanitation. We must look to the welfare of the community as well as of the individual.

Additional reasons have arisen within the past two years. Formerly the unsanitary condition of the cities in the islands belonging to Spain in the West Indies was a constant menace to us. There was always the danger of the introduction of yellow fever because of their close proximity to our shores. Now that we can control conditions in these islands, it will be our own fault if this is repeated. If the American people do their full duty in regard to public health, epidemics of this dreadful disease should become a thing of the past.

From the growing commercial relations with the Phillipine and Hawaiian Islands there comes the additional danger of the introduction of leprosy and the Oriental plague. This latter is a matter that should concern us very greatly at the present moment.

The authorities of our national government have shown wise discretion and a proper appreciation of the necessity for public sanitation in the appointment of General Leonard Wood as governor-general of Cuba. The medical profession points with pride to him as one of its members, and applauds his appointment to such a responsible position. His fame as a civilian and his splendid work as a sanitarian over-shadow even his brilliant service as a soldier.

The Federal government annually expends a large sum of money in maintaining the United States Marine Hospital Service, but it is money well spent, and for which it gets a large return. The care of the sick and disabled seamen becomes of minor importance to the far greater work of watching our ports against the introduction of contagious diseases from without, and preventing their spread from one State to another when they arise within. The great mass of the people, however, do not appreciate what is done by this service in the prevention of disease. As an example, look at the great care which prevented the introduction of cholera into this country from Europe in the summer of 1892, or the almost miraculous prevention of yellow fever when our soldiers were returning from Cuba in the summer of 1898. In each instance some few cases were stopped at New York, but can any one imagine the terrible havoc, the awful loss of life and commerce that would have resulted from an epidemic of either of these diseases in this country? The same care and vigilance are being exercised at present to prevent the introduction of the Oriental plague.

Is it not surprising that so little attention or concern should be paid to the prevention of disease, when any new explosive, a new gun or some torpedo or submarine boat, or any means of warfare is heralded by the newspapers from one end of the country to the other? Surely, one might think our people are more interested in the destruction of life than in its preservation.

England has long been regarded as the birthplace and home of modern sanitary science. In no other country does public medicine receive as much attention, and the benefits are expressed in the low mortality rate. With such a high standard of public health, her methods may well serve as models for other countries. Let us compare the methods and conditions in this country with those of England, and inquire why are we so far behind in the matter of public health?

In the first place, we find men are not trained to be medical officers of health. In some of our large cities there are officers of the health departments who possess the special knowledge necessary for the performance of their duties in some narrow field. In almost every case, however, they have learned from practical experience or special training after their appointment, and were not prepared for the work before they entered upon their duties. Men are not educated in this country with a broad knowledge of public medicine. Indeed, there is only one laboratory of hygiene equipped for giving such instruction, and the demand for such teaching is so slight that there is no systematic curriculum, but only special courses fitted for a few individuals.

What is the explanation of this? The reason is not difficult to find. It is the lack of appreciation by controlling authorities and by the people of the importance of the position of medical officer of health. What is perhaps of more importance is the method by which such appointments are made. Very often the compensation is insufficient to attract men of ability. Aside from that, the uncertainty of the tenure of office in the service of almost every board of health anywhere in this country is sufficient to deter any young man from making special preparation for such a position. Appointments are usually made for political reasons, and not because of a man's fitness for the position. Under such circumstances it becomes impossible for such officers, even if they have the ability, to act in the prompt, firm and fearless manner that is often required of them. Too often the welfare of a community is jeopardized because the selfish interests of a few people with influence are different from those of the greatest number.

People do not realize that a medical officer of health should have a knowledge and training quite different from that required of a practitioner of medicine. Outside the great cities the health officers in this country are usually general practitioners, and all the subordinate officers, such as sanitary inspectors, are appointed solely for political reasons, with absolutely no ability or training for the duties to be performed.

Contrast this with the condition in England. Men who have not taken special instruction and who do not hold a diploma in public health in addition to the diploma in medicine, are ineligible as medical officers of health. Subordinate officers must have taken a course at a training school in public health. The appointment is made because of the fitness and ability of the man and not merely for political reasons. Moreover, the compensation is sufficient to get men of ability, and the tenure of office as long as its duties are properly performed.

How can we remedy our condition? Easily enough. Take the whole matter out of politics. Not only the health officers, for they are often helpless, but the boards of health themselves should be taken out of politics. Let us pattern after New York City, which has the best and most advanced health department in this country. Appoint an equal number of health commissioners from each political party and see that these are not merely politicians, but men who know something of sanitation. Let these appoint the health officer and have every salaried position filled by an impartial civil service examination.

People do not select their family physician because of his "political pull," but because of his ability. Why should they not select the health officer who is to guard the health of the community on the same ground?

If the people will do their duty in attaching due importance to the position of health officer and place it on a high plane, the educational institutions will promptly provide the necessary training to fit them for it. Already some limited instruction is offered for special work in sanitation by the boards of health of Vermont, of the city of New York and of Chicago. There is an urgent need of more public laboratories of hygiene, not only for teaching purposes, but for the prompt and exact recognition and investigation of contagious diseases. The Federal government offers to furnish $15,000 for the equipment of agricultural experiment stations on condition that the State provide a similar amount. With a very few exceptions our State governments do not appreciate what good can come to the people from these laboratories of public health. Are not the lives of the citizens of this great country worth as much as the products of the soil?

Another great trouble in this country is the lack of uniformity both in the laws and in the methods of dealing with the contagious diseases that occur in epidemics. In this respect England is very fortunate in having a central control of all these matters as well as of quarantine. A careful consideration of the unpardonable neglect in dealing with matters of public health in some sections, the differences arising between rival States and cities, and the deliberate attempts to misrepresent conditions as to the existence of contagious diseases, will convince any thoughtful person of the necessity of increasing the scope of the Marine Hospital Service or establishing a national bureau of health having general control of such matters.

You all recall how the whole world was shocked two summers ago at the awful loss of life when the ocean steamer La Bourgogne was wrecked and 560 lives were lost in a few minutes. Those of us who lost friends on that unfortunate vessel were most severe in our condemnation of the management and discipline of the ship. Whatever may have been the excuse for her having been out of her course, many felt that the disaster was preventable; that the awful sacrifice of life was unnecessary and could have been avoided. It is such terrible catastrophes as these that make some people afraid to travel by sea, although they enter a railroad train without any thought of danger. In the aggregate, the loss of life from the latter method of traveling is probably greater than from the former, but we read of it in the papers every day, we get accustomed to it and never stop to think of it, while the loss of life from an accident to a steamship makes us shudder. Did you ever stop to think that one-third of the deaths occurring in this country in 1890 were due to preventable diseases? Think of it, over 250,000 lives sacrificed in one year! Some of these could not have been saved. The infection may in some instances have resulted from circumstances which were uncontrollable, but it is safe to say that at least three-fourths of these deaths could have been avoided had all the knowledge of the cause the way of spreading and the means of prevention been properly used. Is it not time that we do something in preventive medicine? If we are so ready to blame the master of a vessel for the loss of life on an ocean steamer resulting from a collision, should we not blame ourselves with indifference that is unpardonable and neglect that is criminal?

In former ages smallpox was the most dreaded contagion. More than a century ago Jenner gave vaccination to mankind and steadily since then this disease has been shorn of its horrors until the mortality from it at present is comparatively trifling. The great plague of modern civilization is tuberculosis (consumption). It is more to be dreaded now than was smallpox before the days of vaccination, as it causes relatively twice as many deaths at the present time as smallpox did then. In fact it caused more deaths in 1433 cities and towns in the United States, having a population of over 21,000,000, in the year 1898, than all other contagious diseases put together. One death in every nine from all causes in these cities resulted from tuberculosis. If it spread rapidly and ran a short course, the people would demand that every precaution be taken to prevent its spread, but as we see it constantly everywhere about us, it becomes so familiar that we neglect it and cease to fear it. This is the more remarkable when it is remembered that no disease is better understood or its spread more easily prevented. It does not require isolation like the highly contagious diseases. Simple precautions may be applied in the patient's home so that the consumptive may not endanger the lives of those about him.

When one recalls the universal fright, the confusion and anxiety to get away that prevailed among the panic-stricken people of Galveston less than three years ago when it was telegraphed to Washington that yellow fever existed here, it is difficult to understand the supreme indifference that exists among these same people not only to tuberculosis, but to sanitary measures in general. And yet tuberculosis causes more deaths every month in New Orleans alone than occurred in the entire State of Louisiana or in Mississippi from yellow fever in 1898.

I am aware that those who preach the communicability of tuberculosis are considered by some to be extremists and fanatics, but I challenge any one, either in the medical profession or out of it, to prove that tuberculosis can be caused in any other way than by a specific germ or that this germ ever arises from any other source than from some previous case of the disease.

How well do we all remember the Maine! The mere mention of her name fills us with emotion. Whoever destroyed her, the thought of it makes the blood of every American citizen boil with righteous indignation to think 266 lives were needlessly sacrificed by the most cowardly and barbarous act of modern civilization. As a result of the inhuman destruction of life in Cuba, this country was willing to go to war with a foreign power. The results are so recent that it is unnecessary to recall them. The war was very exceptional in the small number of our men killed by bullets, but it was just as remarkable for the large number of deaths from disease.

In the Civil War the number dying from disease was twice the number killed in battle and dying from wounds. That was at a time when the causes, the ways of spreading and the means of preventing camp disease were unknown. In the Spanish-American War the number of deaths from disease was more than five times the number resulting from bullets. The greatest disgrace is that over 2000 of these did not occur in an active campaign, but in the training camps at home, where the sanitary conditions could have been and should have been controlled. Most of these deaths were due to diseases that are well understood and entirely preventable. The fault was not with the medical department of the regular army, but the primary cause of this enormous mortality was the method of making appointments to responsible positions purely for political reasons and not because of fitness for the position.

Contrast with this the conditions in the Philippines, where, notwithstanding the climate and inability to control sanitary conditions —in an active campaign, the mortality from disease for the year 1899 did not exceed the number killed and dying from wounds. Or, contrast the condition of the British army in South Africa, where, up to March 1, 1900, the number of deaths from disease was less than one-half of those killed in battle and less than one-eighth of those wounded.

Without offering any apologies for the mismanagement of our army in the summer of 1898, let me call your attention to the high mortality from typhoid fever constantly occurring among us at home. There probably occur annually in the United States from 25,000 to 30,000 deaths from typhoid fever, probably representing nearly 200,000 cases of the disease. In Philadelphia alone, with a little over 1,000,000 population, there were 7985 cases of typhoid fever in 1899. An English writer has said: "For every case of typhoid fever somebody ought to be hung." Would it not be well for the good people and "yellow journals" who strained so hard at this comparative gnat in the form of mortality rate in our army two summers ago, to concern themselves more about swallowing a camel in the shape of the inexcusably high mortality rate from typhoid fever at home? It is difficult to understand how a people that felt so indignant at the loss of life on the Maine will tolerate such a loss of life from causes that can be prevented. The fault is ours; the way is clear; the certain means of prevention is at hand, it only needs to be applied.

The achievements of modern sanitary science are numerous and striking. The great reduction of mortality resulting from a pure water supply and from a good sewerage system has been experienced by so many cities and towns throughout the country that everyone is familiar with it, and these two matters have become corner stones in the foundation of public sanitation.

As a result of cleanliness enforced by Americans, the mortality of Santiago de Cuba for 1899 was but 30 per cent. of the average annual mortality rate for the three years preceding, notwithstanding an inadequate water supply and the absence of a sewerage system. The mortality for Havana for 1899 was but 44 per cent. of the average for the three years before that time, even without a sewerage system.

However, it is not only in cities that have been so indescribably filthy as these were under Spanish control that great improvements have been made. In New York city, where sanitary matters have always received due attention, there has been a reduction of 25 per cent. in the death rate from 1887 to 1897 without any single great public improvement, such as a purer water supply or better sewerage system. This improvement has resulted chiefly from the thorough and scientific methods used in the recognition, notification and isolation of contagious diseases and the disinfection after their occurrence. Other cities have adopted her methods with the same benefits. This movement has extended so rapidly that at present New York, Boston, Chicago and Philadelphia have medical inspectors of schools to prevent the spread of contagious diseases through the public schools.

Does anyone think the people are not in favor of sanitary improvements? The popular vote in Pittsburg for a pure water supply, the vote in Philadelphia to provide means for purifying the water, and the vote of the property holders in New Orleans for a public sewerage system all indicate that the people are in favor of such improvements, even though they are expensive. It is indeed a sad thing to see the metropolis of the South only taking such action because the recurrence of epidemics affects her commerce. Human life can not be valued in terms of dollars and cents. But it is a still sadder thing to see other cities, like Galveston, equally bad off from a sanitary standpoint, failing to profit by her unfortunate experience.

I hope that each one of you graduating in medicine today will consider it a duty you owe to the State that has given you a free education to lend your best efforts to the cause of public medicine, to the prevention of disease.

As physicians you should keep out of politics, but if the important position of health officer or of membership of a board of health should come to you, do not shirk the responsibility merely because the duties of the position may not have been properly performed in the past. You are fitted for such work, and we want capable, strong and fearless men for such positions, and not mere figureheads, who know nothing of sanitation, and often care even less.

But it is not only as public sanitarians that opportunities are opened to you. Every physician can do much in his private practice toward the prevention of disease. Dr. Holmes once humorously said: "Physicians desire for their patients great longevity, with frequent illness." It is entirely unnecessary to attempt to refute these suggestions before this audience. The question is, however, often asked, "Why should a physician, who makes his living by treating the sick, use his knowledge of hygiene in the prevention of disease? Even from a purely business standpoint it is to his advantage to do so. The time is past when intelligent people are satisfied to have the physician call, prescribe his medicines and then leave. They want to know something of the nature and the cause of the disease, the way in which it was most probably contracted, and, if preventable, the means of preventing its spread. If the physician does not give this information he may soon find himself supplanted by some one else who will.

But there is a higher moral reason why every true physician will practice preventive medicine on all occasions, whether it is of any pecuniary benefit to him or not. It is the duty of the physician to save life, to lessen and prevent suffering whenever and wherever he can, and if any of you have no higher motive in the practice of medicine than the money to be gained you had better give it up right now.

If the public water supply is polluted, you should tell your patients how it may be rendered free from danger. If the board of health or the health officer in the community in which you live should not pay proper attention to the isolation of contagious diseases and disinfection after the occurrence, that does not excuse the attending physician for any neglect of the minutest detail in these matters. In limiting the spread of consumption the attending physician can accomplish far more in instructing the patient how to prevent its communication to others than can be done by the public health officials. Regarding personal hygiene, such, for example, as the evils of over-eating and over-working, which are so common at present, the people can only be educated by their private physicians. Indeed, as educators of the people in matters concerning the public health of the community, as well as the health of the individual, the physician and the public press are the most potent factors. I sincerely trust you will not regard dosing your patients as your only duty and that you will pay due attention to the prevention of disease.

Time does not permit me to address any words especially to the graduates in pharmacy and to the graduates of the school of nursing. No one appreciates more than the physician what valuable allies he has in the nurse and the skilled pharmacist. Indeed, it often happens that the nurse in an emergency or by faithful devotion to duty, contributes more than the doctor to saving the life of the patient. Perhaps the highest compliment that could be paid to the pharmacist in recognition of the dignity and efficiency of his profession is seen in the way most of the State examining boards of this country have within the past decade required all candidates for registration to be graduates of a school of pharmacy.

It does not matter to the true soldier in what branch of the service he fights; he is content to know that he is performing his duty to the best of his ability wherever he may be stationed. You are all today enlisting in a noble cause and I may say one final word of advice which will apply to all, wherever you may serve: Be in earnest in your work and always, under all circumstances, do your full duty. Be true to your profession; be true to your alma mater; be true to the community and State in which you live, and

"This above all; to thine own self be true,
And it must follow, as the night the day,
Thou canst not then be false to any man."

